# Domains of life sciences in spacefaring: what, where, and how to get involved

**DOI:** 10.1038/s41526-024-00354-y

**Published:** 2024-01-29

**Authors:** Aaron J. Berliner, Spencer Zezulka, Gwyneth A. Hutchinson, Sophia Bertoldo, Charles S. Cockell, Adam P. Arkin

**Affiliations:** 1 Center for the Utilization of Biological Engineering in Space (CUBES), Berkeley, CA USA; 2 Department of Bioengineering, University of California Berkeley, Berkeley, CA USA; 3 Program in Aerospace Engineering, University of California Berkeley, Berkeley, CA USA; 4 School of Information, University of California Berkeley, Berkeley, CA USA; 5 UK Centre for Astrobiology, School of Physics and Astronomy, University of Edinburgh, Edinburgh, UK

**Keywords:** Biotechnology, Biomedical engineering, Aerospace engineering, Scientific community, Biogeochemistry

## Abstract

The integration of biology and spacefaring has led to the development of three interrelated fields: Astrobiology, Bioastronautics, and Space Bioprocess Engineering. Astrobiology is concerned with the study of the origin, evolution, distribution, and future of life in the universe, while Bioastronautics focuses on the effects of spaceflight on biological systems, including human physiology and psychology. Space Bioprocess Engineering, on the other hand, deals with the design, deployment, and management of biotechnology for human exploration. This paper highlights the unique contributions of each field and outlines opportunities for biologists to engage in these exciting avenues of research. By providing a clear overview of the major fields of biology and spacefaring, this paper serves as a valuable resource for scientists and researchers interested in exploring the integration of these disciplines.

## Introduction

The imagery from NASA’s Mars 2020 Perseverance Rover has provided an opportunity for our imagination to sojourn to new worlds beyond Earth. As we embark on a journey to push the boundaries of the unforgiving and vastly uncharacterized frontier of space, we must understand how life, including our own species, could survive and thrive in the myriad space environments we will encounter. We will face unprecedented challenges in building a rich biological ecology to provide resources—from clean food and water to pharmaceuticals and materials—to derisk our extended stays off Earth with an emphasis on efficiency and minimizing environmental impact^1,2^. We compare and contrast the major fields for integrating biology and spacefaring—Astrobiology (AB), Bioastronautics (BA), and Space Bioprocess Engineering (SBE)—first by providing a description of what each field entails and highlighting the ways in which they may evolve and then outlining where interested biologists may begin their voyage toward these exciting avenues for study.

## Astrobiology

Astrobiology is the study of the origin, evolution, distribution, and future of life in the universe^3,4^. This interdisciplinary field encompasses the search for habitable environments beyond Earth, the search for evidence of prebiotic chemistry and microbial life on Mars and other bodies in our Solar System, and the study of intelligent life’s potential for emergence elsewhere^5^. Astrobiology also considers the potential impact of extraterrestrial life on human society, our future exploration, and settlement of the universe^6^. One key goal of Astrobiology is to predict and discover habitable environments in our Solar System and beyond^7^ by studying conditions on planets, moons, and other bodies that could support life as we know it. For example, recent evidence for liquid water on Mars^8^ has sparked interest in the potential for microbial life to exist on the Red Planet or even to persist in the subsurface today. Similarly, studies of icy moons, such as those conducted to explore the Jovian moon Europa’s subsurface ocean^9,10^ and the Saturnian moon Enceladus’ geysers of water vapor and ice, which are thought to be connected to a subsurface water body^11,12^ have revealed possible habitats for life^13^. Astrobiologists also seek evidence of prebiotic chemistry and microbial life on other celestial bodies^14,15^. The Curiosity Rover’s detection of organic molecules—potential building blocks of life– suggests there are source materials for life’s origins and possible products from its existence.^16,17^. Similarly, the Cassini mission to Enceladus revealed the presence of molecular hydrogen, phosphorus, and other organics that have been proposed as energetic substrates for sustaining microbial life^18–21^. The possibility of microbial life existing on other planets, coupled with its undeniable presence on Earth, demands careful consideration and mindfulness in our approach to space exploration. Robust planetary protection measures are essential to safeguard our research endeavors. Astrobiologists play a crucial role in developing and executing protocols to prevent both forward and backward contamination events^22^. These measures are vital to preserving the integrity of scientific inquiries into the origins and distribution of life in space. Furthermore, astrobiologists address and study biological spacecraft burdens, aiming to protect spacecraft environments from pathogenic microorganisms that could harm crew members and to avoid those microbes negatively impacting individuals or environments upon return to Earth. Model organisms, such as *Salmonella* and *Serratia marcescens*, have been used aboard the ISS to study and characterize augmented virulence in microorganisms exposed to spaceflight, providing us with insights that allow for more meaningful risk assessments and improved countermeasures^23,24^. Astrobiologists also seek to expand our knowledge base for extremophilic organisms such as Haloarchaea and tardigrades, which can survive in even the most hostile conditions^25,26^. By ensuring the implementation of powerful planetary protection measures, astrobiologists help pave the way for safe and responsible space exploration^22^. Moreover, astrobiologists also explore how intelligent life elsewhere in the universe may arise and how that life may one day affect human society. While the likelihood of intelligent life existing on other planets is currently unknown, it remains a worthwhile pursuit to determine how we may interact with any life forms, intelligent or otherwise, and in what ways they may impact the well-being of our species or Earth’s biosphere^6^. An intersectional topic of Astrobiology and the sociology, law, and ethics of space travel is the definition of the rules and methods of engagement with any extraterrestrial life form we may encounter. How we minimize deleterious effects while allowing for fruitful engagement is challenging on scientific, technological, and humanistic levels. As we continue to explore space in search of suitable environments and evidence of life offworld, we augment our understanding of the requirements for supporting life and the potential for humans to live and establish settlements on other planets. This knowledge will be crucial for planning and executing future space missions, spacefaring, and settlement efforts.

## Bioastronautics

Bioastronautics is the study of the effects of spaceflight on biological systems, including physiological and psychological effects on humans to survive and prosper in extraterrestrial environments^27^. This interdisciplinary domain combines the fields of biology, medicine, and engineering to address the challenges and opportunities space exploration presents. Bioastronautics explores the physiological and psychological impacts on humans resulting from spaceflight and other missions beyond Earth^28,29^. Research demonstrates that extended periods of weightlessness and radiation exposure can have negative effects on the human body, including bone density loss; changes in the immune, neural, and optical systems; (Spaceflight-Associated Neuro-Ocular Syndrome), and increased risk of cardiovascular disease^28,30–35^. Additionally, space missions’ inherent isolation and confinement, coupled with the lack of novelty that accompanies many long-duration missions, can lead to potent psychological challenges, including anxiety, depression, and cognitive decline that may endanger mission success^36,37^. In addition to developing an understanding of the potential repercussions of spaceflight, Bioastronautic specialists also create life support systems and countermeasures to protect astronauts from experiencing those effects^38^. The space environment presents unique challenges for maintaining the health and well-being of astronauts, including exposure to radiation, microgravity, and isolation, prompting the need to develop and test life support systems such as air and water recycling systems to sustain the crew during long-duration missions^39–41^. To gain profound insights into these phenomena, Bioastronautics leverages a diverse array of organisms as invaluable data sources, enabling a comprehensive understanding and forming a launching pad for countermeasure development for the impacts of spaceflight environments. This encompassing approach includes the study of humans, mice, insects, plants, and microbes alike along with other less common model species such as apes, squid, and tortoises. The increasing numbers of people dedicated to establishing a spacefaring future are also driving research into how variables such as age, gender, genetics, and lifetime environmental exposure can lead to varied responses and differential health outcomes in the space environment (and lingering effects afterward)^42,43^. As larger, more diverse groups spend longer durations offworld, space communities will begin to emulate extended societies, necessitating deeper study into the formation of these societies, their norms, and how best to develop societal structures^44,45^.

Space biology predominantly employs model organisms to understand the biological effects of spaceflight and to explore fundamental biology and medical questions. Rodent models, such as those in NASA’s Rodent Research and JAXA’s Mouse Habitat Unit series, are crucial for insights into human health in space. Zebrafish, medaka fish, fruit flies, and worms are used to study the impacts of microgravity and space stressors^43^. Plant models focus on space agriculture and gravitropism, essential for food production and oxygen renewal. Microbes are studied for their roles in human microbiomes, plant-microbe interactions, and spacecraft environmental cleanliness, contributing to fields like astrobiology and space biotechnology. These model organisms provide vital data for understanding and adapting to the challenges of space travel.

In recent years, there has been growing interest in studying the microbiome of astronauts due to the unique environment of space, which can impact microorganismal composition and function^46^. Microbiomes vary widely depending on age, environment, genetics, disease state, medication intake, age, and other factors pertaining to the host, and these differences may be even more salient in space^47^. Research has demonstrated that spaceflight induces significant alterations in host-associated microbiomes, engendering negative effects on both astronaut and plant health, including a heightened risk of infection, impaired immune function, and diminished biosystem operation^48^. To address these challenges, one potential mitigation strategy involves the use of pre- and probiotics, dietary supplements that help maintain microbiota balance^49–51^. The physiological impacts of spaceflight on the human body extend to microorganisms, both within astronauts themselves and potentially in the spacecraft environment. Microbes respond differently in space compared to terrestrial conditions, leading to notable effects on various functional aspects, such as cell physiology, gene expression, community diversity, antibiotic resistance, differentiation, biofilm formation, host-pathogen interactions, and overall virulence^52,53^. The alterations in the gut microbiome, dysregulation of the immune system, and increased microbial pathogen virulence all pose significant risks for mission failure^54^. Consequently, Bioastronautics emphasizes the crucial need to understand and address these microorganismal changes to ensure the well-being of astronauts and the overall success of space missions.

Another approach is to design spacecraft and space habitats with microbial control measures—such as air and water filtration systems—to reduce the likelihood of introducing potentially harmful microorganisms^55–58^. Such integration between microbiome exploration and engineering is also of interest in SBE efforts such as ESA’s MELiSSA (Micro-Ecological Life Support System Alternative) and NASA’s CUBES (Center for the Utilization of Biological Engineering in Space) programs^59–61^. Bioastronautics research has proven valuable in combating disease states in the Earth-bound population, an application for which cancer diagnosis and treatment provide a prime example^62^. Additional dual-use benefits arise from cross-cutting Bioastronautics research in telemedicine, portable ultrasounds, and rapid pandemic response^63–66^.

## Space bioprocess engineering

Harnessing biotechnology for space as a national need was perhaps first articulated in a 1992 National Academies Report “Putting Biotechnology to Work.”^67^. As the timelines for longer duration and deeper space missions become shorter, the field of Space Bioprocess Engineering (SBE) has become better defined. While still nascent even now, SBE is the multi-disciplinary approach to design, realize, and manage a biologically-driven space mission as it relates to addressing NASA’s Space Technology Grand Challenges^1,68^. SBE aims to advance technologies to support the nutritional, medical, environmental, and incidental material requirements that will sustain astronauts against the harsh conditions of interplanetary transit and habitation offworld^61,69^, especially in cases where supply chains and other support from Earth are limited. SBE focuses on developing and optimizing bioprocesses for use in space, while Bioastronautics focuses on studying and addressing the effects of spaceflight on living organisms. SBE combines synthetic biology and bioprocess engineering under extreme conditions to enable and sustain a biological presence in space^2^. SBE technologies^70^ generalize into categories for in situ resource utilization (ISRU)^71,72^, loop closure (LC)^73^, in situ manufacturing (ISM)^74^, and food and pharmaceutical synthesis (FPS)^75–78^. This includes the development of ultra-efficient and regenerative resource capture and recycling systems, such as carbon and nitrogen capture, and water reclamation^79^. SBE also includes efforts to define novel routes to “self-repairing”, “growable”, and “self-driving” bioprocessing infrastructure. This includes programmable, responsive, and scalable biomanufacturing processes for the production of materials, chemicals, and pharmaceuticals, as well as functional foods with configurable nutrient profiles^74^. Moreover, SBE focuses on the creation of resilient and adaptive platform organisms that can thrive in low-resource, high-stress environments. This includes innovations that powerfully incorporate and utilize both microbes and plants capable of augmenting biomanufacturing efforts and life support potential^80^. SBE’s pursuit of ambitious goals encompasses a vast array of microbial species, including *Athrospira platensis*, a cyanobacterium that is being built as a platform organism for the production of biosynthetic pharmaceuticals and nutrients; *Sphingomonas*; *Methanobacterium thermoautotrophicum*; and *Cupriavidus necator* a soil-dwelling bacterium being used for bioplastics production just to name a handful^2,61,81^. Furthermore, this emerging discipline embraces the indispensable contributions of higher-order plants, such as lettuces and potatoes, amplifying the scope and impact of pioneering research in biomanufacturing^77^. At the leading edge is the engineering of other pieces of living infrastructure, such as self-healing materials^82^. These organisms will play an essential role in enabling the production of life-critical, efficient, and sustainable functions for deep space and Earth while maintaining high agency and low risk^74^. However, issues of safety and reliability in the space environment, along with containment and threat of environmental contamination, remain open challenges. The recent release from the National Academies and NASA in the form of “Thriving in Space: Ensuring the Future of Biological and Physical Sciences Research: A Decadal Survey for 2023–2032” recommends that NASA should initiate two research campaigns in the area of SBE over the next ten years^83^. One such campaign, BLiSS (Bioregenerative Life Support Systems), aims to comprehensively understand biological systems to produce food, purify air and water, and manage waste, facilitating long-term space habitation independent of Earth. Recognized in NASA’s technology roadmap, sustainable bioregenerative life support is essential for long-duration missions to preserve health and well-being. The initiative not only promises technological advancements for space exploration but also offers valuable sustainable solutions for Earth.

## Where should biologists start and how can they get involved?

**Fig. 1:**
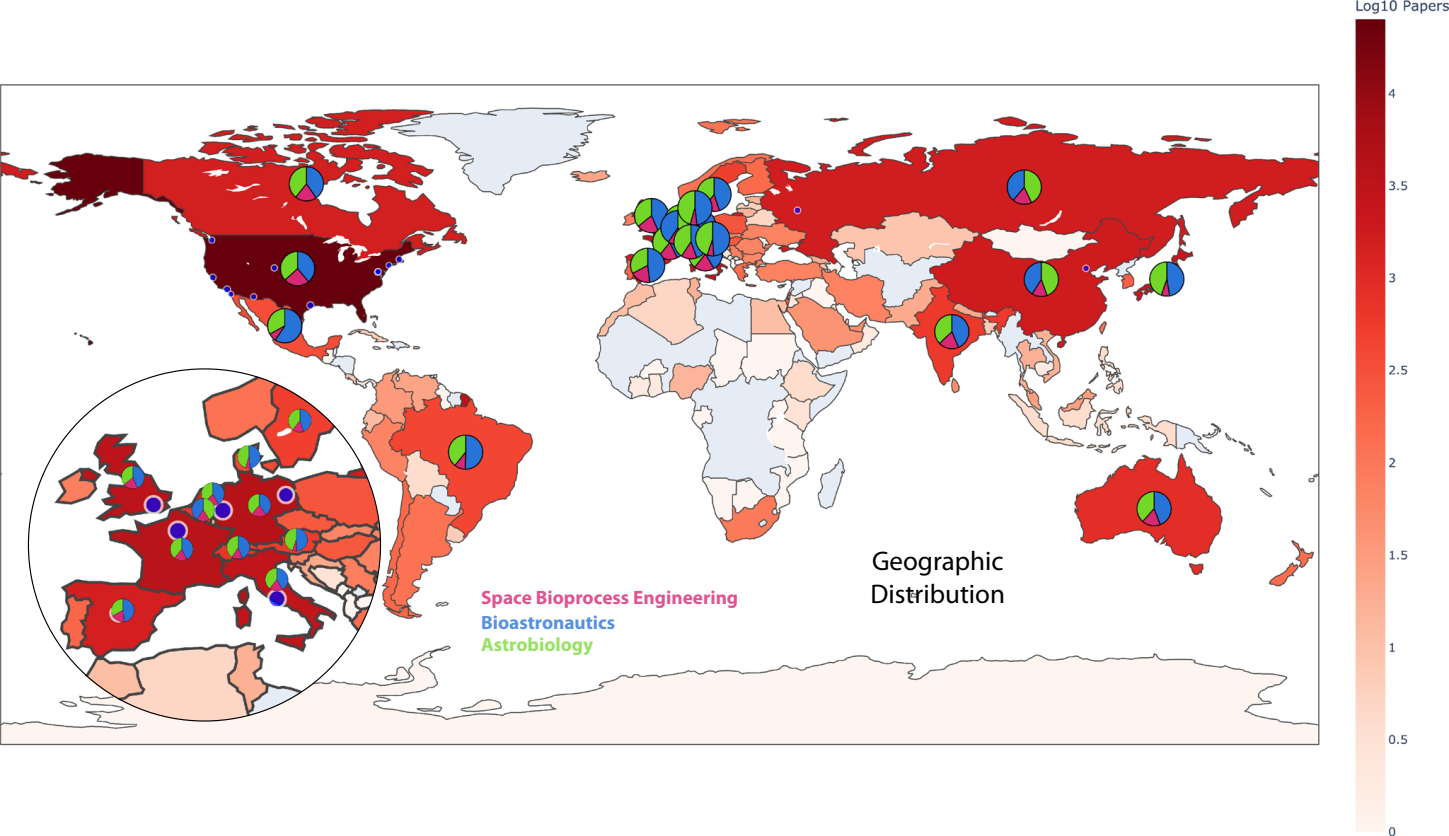
Space biosciences institutional and research data. Geographical distribution of space life science research. Paper count is colored on a log scale in red for all countries available in the bibliometric analysis. Scattered blue dots are marked for cities with the largest citation counts. For the top 20 countries, we show the breakdown of citations as a pie chart in terms of the three corresponding disciplines of Astrobiology (green), Bioastronautics (blue), and Space Bioprocess Engineering (pink). Countries with light blue background indicate zero records. For fig. 1, We show the breakdown of citations as a pie chart in terms of the three corresponding disciplines of Astrobiology (green), Bioastronautics (blue), and Space Bioprocess Engineering (pink). Countries with non-zero records are log-scaled by the color Red. Countries with light blue background indicate zero records.

**Fig. 2:**
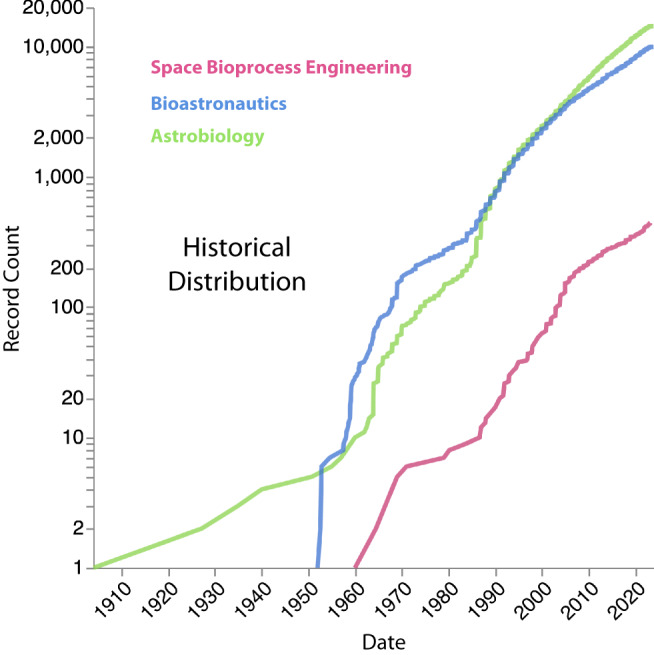
Space biosciences institutional and research data. Historical count of Astrobiology (green), Bioastronautics (blue), and Space Bioprocess Engineering (pink) publications over the past century. For Fig. 2, we show the historical count of Astrobiology (green), Bioastronautics (blue), and Space Bioprocess Engineering (pink) publications over the past century. These colors match those in Fig. 1.

Biologists have many routes to get involved in Astrobiology, Bioastronautics, and/or Space Bioprocess Engineering. In an effort to help facilitate interested biologists, we provide a map in Fig. 1 showcasing the geographical distribution for the three key disciplines. It is evident that research in these disciplines is distributed across several regions, with notable concentrations in North America, Europe, and parts of Asia. Fig. 2 provides a temporal perspective on the growth of these disciplines by displaying the cumulative count of publications over the past century. Astrobiology has had the longest consistent increase in publications, followed by a boom in Bioastronautics as crewed missions arose in the 50s which has since leveled off to match the rate of Astrobiology publications. Space Bioprocess Engineering has had a slower increase since those early days but should rise as missions become longer and in deeper space. This could be attributed to the interdisciplinary nature of Astrobiology, which often necessitates collaborations between researchers from diverse backgrounds such as biology, astronomy, and geology. Although, we note that BA and SBE are also very interdisciplinary.

Table 1 shows the (co-)occurrence of the most commonly author-assigned keywords of interest within their discipline(s). Each publication can have any number of keywords, but each keyword can only be associated once per paper. In this context, the keywords refer to common themes or topics of research interest within each discipline, and their respective prevalence may be used as a proxy for their level of importance or focus in each field. The scaled prevalence column aggregates the overall prominence of each keyword in the combined data from all three disciplines and is used as the metric to determine which keywords to present in this snapshot of topics most relevant to space biosciences. A complete view of this data can be found in the SI.

**Table 1:** Count *ν* and prevalence *P* [%] of top 20 author keywords.

*k*	*ν*_*AB*_(k)	*P*_*AB*_(k) [%]	*ν*_*BA*_(k)	*P*_*BA*_(k) [%]	*ν*_*SBE*_(k)	*P*_*SBE*_(k) [%]	*P*_*S*_(k) [%]
Astrobiology	2234	4.49	26	0.09	10	0.93	1.84
Microgravity	19	0.04	1260	4.56	4	0.37	1.66
Mars	717	1.44	85	0.31	18	1.68	1.14
Origin of life	1497	3.01	0	0.0	1	0.09	1.03
Spaceflight	12	0.02	660	2.39	1	0.09	0.83
Prebiotic chemistry	657	1.32	0	0.0	0	0.0	0.44
In situ resource utilization	2	0.0	8	0.03	13	1.21	0.42
International space station	31	0.06	166	0.6	5	0.47	0.38
Moon	62	0.12	71	0.26	7	0.65	0.34
Space exploration	29	0.06	62	0.22	8	0.75	0.34
Cyanobacteria	62	0.12	8	0.03	9	0.84	0.33
RNA world	442	0.89	0	0.0	0	0.0	0.3
ISRU	2	0.0	8	0.03	9	0.84	0.29
Synthetic biology	52	0.1	3	0.01	8	0.75	0.29
SETI	419	0.84	1	0.0	0	0.0	0.28
Space	19	0.04	126	0.46	3	0.28	0.26
Weightlessness	0	0.0	211	0.76	0	0.0	0.25
Equivalent system mass	0	0.0	3	0.01	8	0.75	0.25
Extremophiles	137	0.28	1	0.0	5	0.47	0.25
Space medicine	2	0.0	174	0.63	1	0.09	0.24

Scopus contains metadata on keywords associated with each publication by their author(s). The top 20 author keywords *k* in the space bioscience corpus are sorted in descending order by scaled prevalence *P*_*S*_(k) within the combined space bioscience corpus, normalized by discipline such that each discipline is of equal importance, preventing the newer fields of BA and SBE from being drowned out by the elder AB. Count is synonymous with multiplicity *ν*_*D*_(*k*) for keyword k in discipline corpus *D*, where *D* ⊂ *S* is the multi-subset of keywords associated with a discipline within *S*, the multi-set containing all keywords in the space bioscience corpus and comprised of the disciplines Astrobiology, Bioastronautics, and Space Bioprocess Engineering, such that *D* ∈ {*AB*, *BA*, *SBE*} and *S* = *AB* + *BA* + *SBE*. Prevalence is defined as the percentage ${P}_{D}(k)\equiv \frac{{\nu }_{D}}{| D| }\times 100 \%$ of which D is comprised of k. Scaled prevalence is defined as ${P}_{S}(k)\equiv {\sum }_{D\in \{AB,BA,SBE\}}\frac{{P}_{D}(k)}{3}$, in which the scaling factor of 1/3 is applied to ensure that ∑_∀*k*_*P*_*S*_(*k*) = 100%. The discipline multi-subset sizes ∣*AB*∣, ∣*BA*∣, and ∣*SBE*∣ are 49792, 27650, and 1071, respectively, for a total multi-set size ∣S∣ of 78513. Author keywords were stripped of white space and decapitalized to ensure degeneracies between semantically-identical, format-incongruous keywords (e.g., “Astrobiology” and “astrobiology“. Note that this does not compress semantically similar abbreviations into their parents (e.g., ISRU, in situ resource utilization).

Of the three space biology domains we have delineated here, the only one that appears as itself is Astrobiology. It is, sensibly, most prevalent in Astrobiology, but is interestingly also relatively well-represented in SBE, which may indicate that studies within the emergent field of Space Bioprocess Engineering may draw upon the better-established discipline of Astrobiology for inspiration and relevance. ‘Microgravity’, on the other hand, has a relatively low count and prevalence in AB, but is highly prevalent in BA. This suggests that the effects of microgravity are a significant focus within BA, probably due to its relevance to human spaceflight and the physiological effects on astronauts, their biological support systems, and life brought to space for experimental purposes. ‘Mars’ is the keyword with the highest product of marginal prevalence, indicating it is a focus for all three domains. This reflects Mars’s significance as a site of search for extraterrestrial life (a core interest in AB), the frequency with which Mars is targeted for human settlement (an interest of SBE), and the concomitant challenge of human transportation to Mars (relevant to BA but perhaps a lesser focus relative to other human spaceflight concerns).

**Fig. 3:**
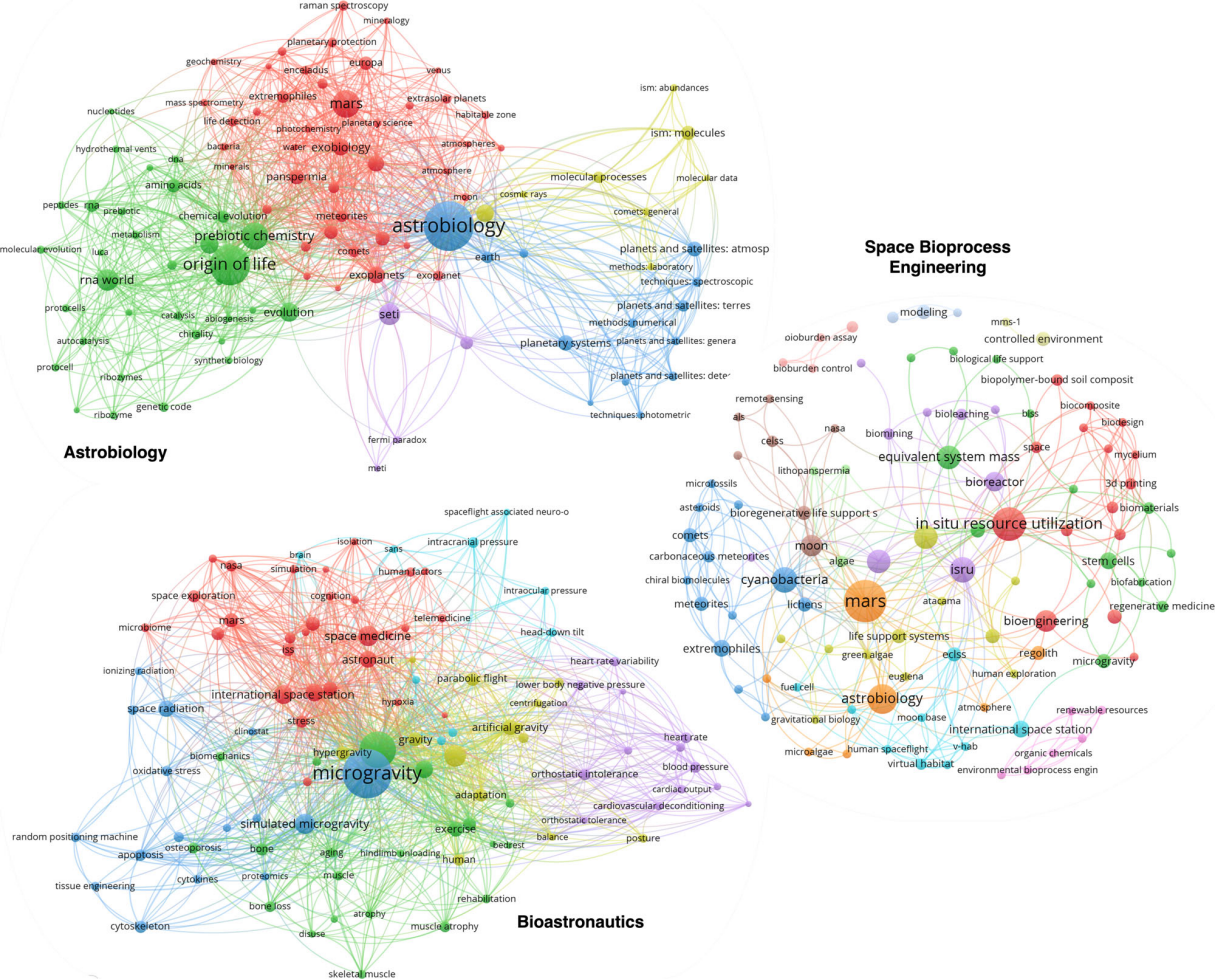
Keyword co-occurrence network graphs (via VOSviewer https://www.vosviewer.com/) for visualizing the subareas within astrobiology, bioastronautics, and space bioprocess engineering. The full research corpus for each discipline was exported directly from Scopus in RIS format after input of the corresponding query. The RIS files were exported including metadata on document title and corresponding author keywords. The bibliometric visualization software VOSviewer was then used to convert this data into a network graph, containing the top 100 most frequently occurring keywords for each discipline. Keyword and corresponding node size scale with its prevalence within the visualized discipline. The nodes and edges of the network were colored automatically according to VOSviewer’s clustering algorithm, with each color representing a distinct cluster---nodes within a cluster co-occur more, implying that the publications they are found in share foci. *n*_*AB*_, *n*_*BA*_, and *n*_*SBE*_, where *n*_*D*_ represents the number of clusters for discipline *D* are 5, 6, and 12, respectively. For Fig. 3, the nodes and edges of the network were colored automatically according to VOSviewer’s clustering algorithm, with each color representing a distinct cluster---nodes within a cluster co-occur more, implying that the publications they are found in share foci. These colors do not match those in Fig. 1 and 2.

The table contains additional intersectional keywords such as ‘international space station’, ‘moon’, ‘space exploration’, and ‘cyanobacteria’, which are shared between all three disciplines, albeit with varied degrees of prevalence. This reflects the common ground and interdisciplinary between AB, BA, and SBE, as all three involve research in space environments (whether Earth-orbiting, lunar, or on a broader scale), and cyanobacteria could be relevant to the study of life in space, space habitat development, and bioengineered systems. Some keywords are completely absent from some disciplines, but still make the top 20 by scaled prevalence. “RNA World“, “SETI“,“Origin of Life“ and “Prebiotic Chemistry“ have high prevalence in AB but are (practically) absent in BA and SBE. This suggests that these topics are predominantly of interest to Astrobiology, dealing with the universal questions about life’s existence and evolution, and less pertinent to the practical, human-centered considerations of BA and the process-focused goals of SBE. Similarly, the keywords “spaceflight”, “weightlessness”, and “space medicine” appear commonly in the keyword corpus for BA but almost never in that for AB and SBE, reflecting the anthropocentric specificity of Bioastronautics, while the keywords “equivalent system mass”, “in situ resource utilization”, and “ISRU” appear in the corpus of SBE but not in that of AB or BA, reflecting the systems engineering focus of SBE. Each discipline has its particular emphasis—the origins and potential of life in the universe for AB, the physiological and psychological effects of spaceflight on humans for BA, and the optimization of bioprocesses for space environments in SBE. Nevertheless, it can be seen from Table 1 that there is a significant degree of common interest across these three fields, reflecting the eclectic nature of space biology. Finally, it is worth noting that the large number of unique author keywords, 29,254, which is as a proportion of all keywords close to 37%, and the small-scaled prevalence of 1.84%—for the keyword of maximum scaled prevalence, “astrobiology”—is indicative of a vast diversity of topics covered by the space bioscience domains. An alternative representation of the domains was derived as Fig. 3. This network visualization shows keyword prevalence, as well as co-occurrence within the respective domains’ bibliographies.

Previous analysis of bibliometric data for Astrobiology^84^ has rendered detailed conclusions on the clustering of topics in that discipline. The query used in the previously-cited work, “astrobiology” itself, renders a similar network graph to that seen in Fig. 3 based on the constructed AB query used here. On this Astrobiology corpus, default VOSviewer settings for top 100 keywords reveals five (rather than the six from the previously mentioned work) clusters. In this context, we can briefly interpret the clusters, which are organized differently but affirm similar conclusions. Cluster 1AB, green, containing terms like “prebiotic chemistry” and “origin of life”, describes the research area within Astrobiology which is concerned with the emergence of life and the mechanisms behind it. Cluster 2AB, purple, contains terms related to our search for life in the universe, like “SETI” and “fermi paradox”. Cluster 3AB, red, contains terms relating to the current theoretical constraints on habitability and the motility of life, such as “habitable zone”, “panspermia”, and “atmospheres”, and regions which are suspected to be habitable, such as “extrasolar planets”, “enceladus”, and “europa”. Cluster 4AB, blue, is focused on the observational and computational technology for contemporary astrobiology. Cluster 5AB, yellow, overlaps to some degree with cluster 3AB, but seems to focus on the space between stars (e.g., “ism: abundances”, “ism: molecules") and the chemical processes possible there.

The network diagram for Bioastronautics is divided into six clusters. Microgravity, the most common term, is centralized, as expected from Table 1. It is classified by the VOSviewer algorithm into cluster 1BA, which contains research ostensibly related to analyzing and mitigating effects of space ("microgravity”, “simulated microgravity”, “ionizing radiation”, “oxidative stress”) and the biological response (“tissue engineering”, “oxidative stress”, “apoptosis"). Cluster 2BA, green, is similarly related to the effects of spaceflight (“spaceflight”, [the largest green dot, close to the center, with keyword omitted] “, but contains more entries relating to kinesiology ("exercise”, “biomechanics”, “muscle atrophy") and tissue science ("bone”, “osteoporosis”, “skeletal muscle"). Cluster 2BA might be distinguished from Cluster 1BA by an increased concentration of research interests that are more easily generalizable to medicine on Earth and can be experimented with more easily on Earth. Cluster 3BA, red, is focused on the astronaut as an individual human being (“astronaut”, “human factors”) and relates more to logistic support and sustainability (“cognition”, “telemedicine”, “space medicine”, “international space station”). Cluster 4BA, cyan, is specifically focused on the brain and neurology (“brain”, “intracranial pressure”), while cluster 5BA, purple, is focused on cardiovascular effects (“orthostatic intolerance”, “blood pressure”, “heart rate variability”). Cluster 6BA, yellow, seems to be focused on simulating (a lack of) gravity ("artificial gravity”, “hypergravity”, “parabolic flight”), as well as the human consequences ("posture”, “human", “balance”, “adaptation”).

The network for SBE is the hardest to interpret—the clustering algorithm struggles to find relations and cluster research areas within the corpus as robustly as for the other two domains due to the small size of the corpus. Indeed, for far fewer publications, it finds far more clusters–12, in this case. While this might change in the future, the clearest conclusion that can be drawn from the clusters is that they are unclear and suspiciously organized. Indeed, the clusters are varied and nonconforming enough that their interpretation is left to the reader. It is at least corroborated by the network graph representation; however, the highly prevalent keywords (“mars”, “in situ resource utilization”, “equivalent system mass”) tend to also be highly centralized visually, implying that they are also central to the field topically.

A common factor across these space biology domains is the special context of operating in an offworld environment. Biologists can play an important role in filling gaps in our understanding of the potential for life on other planets and moons, as well as addressing unknown impacts of space environments on living organisms. Biologists can also help to develop new techniques and technologies for detecting biosignatures of life in the universe and for growing plants and other organisms in space—each offering new avenues to consider biological questions in a new context. For biologists interested in exploring Astrobiology, Bioastronautics, and/or Space Bioprocess Engineering, there are several conferences and symposiums that can provide learning and networking opportunities. Some examples include the Astrobiology Science Conferences (ABSciCon), the American Geophysical Union (AGU) meetings, the Lunar and Planetary Science Conferences (LPSC), the American Society for Gravitational and Space Research (ASGSR) conferences, the International Conference on Environmental Systems (ICES), the American Chemical Institute’s (AIChE) Adaptive Research and Technologies from Biological and Chemical Engineering (STAR Tech), and the newly founded ASCEND conferences. Interested students should also explore the offerings from the Committee on Space Research (COSPAR), which include a platform for all scientists to converse about issues that could influence scientific research in space. These forums provide opportunities for researchers and scientists from a variety of disciplines to come together to share ideas and collaborate on projects.

Active collaboration with established organizations and engagement in a consortium can offer mentorship, collaborative opportunities, and avenues for acquiring knowledge. Interested students should explore the Open Science Working Groups under the aegis of NASA, which provide an effective platform for individuals to play an active part and gain insights from ongoing projects in open-source science. Their commitment to promoting an open exchange of ideas, collaboration, and dissemination of data and insights makes them an instrumental resource for those eager to make significant contributions to open-source science. In a similar vein, the European Space Agency (ESA) facilitates Topical Teams, which are clusters of experts focused on various segments of space research that afford interested students and researchers an opportunity to engage with and learn from professionals across different space-related fields. The Japan Society for Space Biology offers opportunities to enrich one’s understanding of space biology while contributing to the field’s growth. This society is dedicated to furthering research in space biology, promoting scholarly interaction among researchers from various disciplines. NASA’s Human Research Program (HRP) organizes investigator meetings, providing a valuable forum for researchers to showcase their work, garner feedback, and engage with other investigators. For those residing in Europe, the European Low Gravity Research Association (ELGRA) presents a multitude of opportunities. ELGRA is devoted to fostering scientific research in environments with reduced gravity, creating a dynamic platform for intellectual exchange among scientists and engineers. Participation in these groups not only enhances your expertise and abilities but also expands your scientific community, paving the way for new collaboration opportunities.

## Moving forward

Over the ensuing two decades, our world is poised to embark on a series of more frequent and extended missions in near-Earth space and beyond, culminating in the ambitious prospect of a crewed expedition to Mars lasting potentially over four years. This underscores a pressing demand for a skilled and dedicated workforce capable of addressing the multifaceted challenges that lie ahead. As humanity’s presence in space endures, it becomes increasingly evident that their well-being is intricately intertwined with the symbiotic relationships they share with their microbiomes and the plant life integral to their regenerative life support systems and sustenance. Navigating the web of interactions and intricacies that govern the long-term implications for these organisms and the ecologies they collectively form represents a formidable scientific and technological undertaking in the realm of Bioastronautics.

The duration of space sojourns directly correlates with the necessity for crucial services encompassing waste recycling and the bioproduction of essential commodities such as food, pharmaceuticals, and high-value chemicals. These resources, which would otherwise entail exorbitant expenses for transportation and storage, must be produced reliably and efficiently to accommodate the temporal and quantitative demands of burgeoning populations. Within this context, the field of Space Bioprocess Engineering must grapple with the intricate task of establishing predictable, stable, and efficient methodologies to facilitate programmable biosynthesis of requisite products, while concurrently devising strategies to pre-process and harness waste streams for mission viability.

Moreover, as our observational instruments attain heightened precision and humanity extends its frontiers into the cosmos, a compelling impetus arises for the identification of potential extraterrestrial life forms range from primitive to advanced. Such a pursuit necessitates a proactive and strategic approach, allowing us to foster constructive engagement with these potential life forms. Recent testimonies to legislative bodies even suggest the intriguing possibility that alien life may have reached us first. In light of this, astrobiologists armed with the expertise to discern and comprehend these novel forms of life assume a role of paramount significance, poised to illuminate the enigmatic aspects of existence beyond our terrestrial realm. Together, these fields represent a multifaceted approach to understanding and exploring the potential for life beyond Earth and an exciting opportunity to create and train a new multi-disciplinary workforce to guide our continued explorations beyond Earth^70^.

## Methods

Bibliometric analysis was conducted to compare the fields of Astrobiology (AB), Bioastronautics (BA), and Space Bioprocess Engineering (SBE). The methods used in this analysis are detailed in Boxes 1-3 below.

### True positive sets

The authors compiled papers known to belong to the body of work corresponding to each domain of space biosciences from trusted sources, hereafter referred to as the sets of true positives. To assemble a set of true positives for each field, multiple sources were chosen for each space bioscience to ensure diverse coverage from the queries. The true positive sets for AB, BA, and SBE contained 23, 27, and 14 papers, respectively. These sets are available for download in the Supplementary Information (SI). The true positives for each field were then split 30–70 (70% training data, 30% validation) via scikit-learn into training and validation sets.

### Query construction and refinement

The authors then interfaced with the Scopus API to construct subqueries from which to derive performance metrics using an original Python library. The Python library allowed for a keyword, title, and abstract extraction from the training set. The authors based their subquery engineering on this text. The recall for each query in both the training and validation sets was calculated programmatically via the library by dividing the number of true positives covered by the query by the total number of true positives in the training set. After each subquery was tested, the library provided the training recall and identified which documents from the training set were and were not returned by the query. For each discipline, the authors iterated on the corresponding query until they reached the recall threshold for the validation set. Final queries can be found in the SI.

### Search execution and manual evaluation

For the same discipline, the authors entered the finalized queries into the Scopus search. The results were sorted by citations, and the top 50, bottom 50, and a random sampling of 50 in between were manually evaluated by the authors to check for false positives. The authors set a precision threshold within the scope of the paper of 0.8 (Table 2) and iterated on an exclusion step to increase precision in the queries. After each exclusive query iteration, the query was inputted to the library to check for any inadvertent decrease in recall. This was repeated until the precision threshold was reached without compromising recall below the threshold.

**Table 2:** Calculation of query performance metrics, where *F* ≡ False, *T* ≡ True, *P* ≡ Positive(s), *N* ≡ Negative(s).

Metric	Formula	Threshold
Recall	$\frac{TP}{TP+FN}$	0.8
Precision	$\frac{TP}{TP+FP}$	0.8

Box 1 Astrobiology data was gathered using the query ‘TITLE-ABS-KEY (astrobiolog* OR cosmobiolog* OR exobiolog* OR bioastronom* OR ((extraterrestrial OR exoplanet OR enceladus OR europa) AND habitab*) OR *panspermi*) OR KEY ("origin of life” OR “RNA world” OR “DNA world” OR “prebiotic chemistry” OR “extant life detect*” OR “*extraterrestrial life” OR seti)’

### Data cleaning

The results of the query were then loaded from the Pybliometrics interface to the Scopus API into the notebooks through the standard Python data manipulation modules (Pandas and GeoPandas). Location data was aggregated by country and geocoded using GeoPy and Photon. The aggregated and labeled geospatial table was then joined to a GeoJSON from the gpdvega library containing shape data for the corresponding countries. This data was used to create the choropleths. The Scopus database records keywords corresponding to each publication. The keywords are indeed not words per se but phrases of arbitrarily many words. The phrases were thus decomposed into their constituent parts by splitting each phrase across spaces if the number of characters in the phrase exceeded the longest contiguous single keyword in the dataset.

Box 2 Bioastronautics data was gathered using the query ‘TITLE-ABS-KEY("bioastronautics” OR “space medicine” OR “medicine in space”) OR (TITLE-ABS-KEY(*astronaut* OR human* OR *cosmonaut*) AND (TITLE-ABS-KEY(spaceflight OR microgravit* OR “artificial grav*” OR “*space station”) OR KEY(martian OR lunar)) AND TITLE-ABS-KEY("life science*” OR health OR medic* OR physiolog* OR psycholog* OR vestib* OR ocula* OR cardio* OR cardiac OR nervous OR drug* OR biol* OR immun* OR lymph* OR respirat* OR digestiv* OR reproductive OR “functional resilience”)) AND NOT (TITLE-ABS-KEY("moon face” OR “moon et al” OR “microgravity bioreact*” OR “lunar new year” OR “lunar densito*") OR TRADENAME(lunar))’

Box 3 Space Bioprocess Engineering data was gathered using the query ‘TITLE-ABS-KEY ("space systems bioengineering” OR “space bioprocess engineering” OR “space exploration medical foundry” OR “equivalent system mass” OR (("biomineralogy” OR “cyanobacteria”) AND “asteroid”) OR (("pharmaceutical foundry” OR “photosynth* engineer*” OR “biotech*” OR “bioengineer*” OR “biomanuf*” OR “biological eng*”) AND (mars OR moon OR leo OR “*earth orbit”))) OR KEY (("in situ resource utilization” AND “*bio*”) OR (("radiotroph*”) AND “space radiation”)) OR TITLE ((*bioengineer* OR “biological engineer*”) AND space) AND NOT (TITLE-ABS-KEY (lunate OR “mobile application* rating scale*” OR “Molecular Adsorbents Recirculating System” OR “Matrix attachment regions” OR (mars AND immunodepletion)) OR AUTHLASTNAME(moon))’

### Visualization

The Plotly visualization library was used in conjunction with the Pybliometrics interface to the Scopus API to build the map of Fig. 1. The Altair visualization library was used to create the line chart in Fig. 2. The line chart provides a view of how these three disciplines of space life sciences have developed and an idea of their trajectory. The keyword co-occurrence network graphs in Fig. 3 were created via VOSviewer https://www.vosviewer.com/).

### Reporting summary

Further information on research design is available in the Nature Research Reporting Summary linked to this article.

## Supplementary information

Supplementary material includes the compilation of the organization data from Table 1, Figs. 1 and 2, as well as interactive versions, and can be found in the GitHub repository here: https://github.com/spencerzezulka/space_biosciences.

### Supplementary information

Reporting Summary

## Data Availability

All data can be freely accessed in the Github repository here: https://github.com/spencerzezulka/space_biosciences.

## Code availability

All code can be freely accessed in the Github repository here: https://github.com/spencerzezulka/space_biosciences.
